# Heart Replacement Therapy in Young Patients: A Comparative Analysis of HeartMate 3 LVAD and Heart Transplant Using MOMENTUM 3 and UNOS Registry

**DOI:** 10.1016/j.jchf.2026.102948

**Published:** 2026-02-07

**Authors:** Nir Uriel, Gabriel T. Sayer, Boaz Elad, Justin A. Fried, Jayant K. Raikhelkar, Dor Lotan, Daniel J. Goldstein, Ulrich P. Jorde, Joseph C. Cleveland, Mandeep R. Mehra, Stavros G. Drakos, Katherine L. Wood, John D. Henderson, Fei San Lee, Manreet K. Kanwar, Kevin J. Clerkin

**Affiliations:** a Columbia University Irving Medical Center, New York, New York, USA; b lbert Einstein College of Medicine, New York, New York, USA; c Montefiore Medical Center, Bronx, New York, USA; d School of Medicine, University of Colorado, Aurora, Colorado, USA; e Brigham and Women’s Hospital, Boston, Massachusetts, USA; f School of Medicine and University of Utah Health, University of Utah, Salt Lake City, Utah, USA; g University of California, Irvine Medical Center, Orange, California, USA; h Abbott Laboratories, Abbott Park, Illinois, USA; i Department of Medicine, University of Chicago, Chicago, Illinois, USA.

**Keywords:** advanced heart failure, adverse event(s), heart transplantation, left ventricular assist device, propensity score matching, survival

## Abstract

**BACKGROUND:**

Younger patients (18–49 years of age) with advanced heart failure (HF) face unique challenges in decision-making for advanced HF therapies. Although heart transplantation (HT) offers excellent survival, it is associated with finite graft longevity. The HeartMate 3 (HM3) left ventricular assist device (LVAD) has demonstrated promising outcomes, but direct comparisons with those listed for or undergoing HT in this age group remain limited.

**OBJECTIVES:**

This study sought to compare survival and adverse event (AE) outcomes between younger patients receiving HM3 LVAD support and those listed for or undergoing HT.

**METHODS:**

The authors analyzed patients 18–49 years of age from the MOMENTUM 3 (Multicenter Study of MagLev Technology in Patients Undergoing Mechanical Circulatory Support Therapy with HeartMate 3) portfolio (HM3 cohort; n = 420) and the UNOS (United Network for Organ Sharing) registry (HT listing cohort; n = 1,955) (HT recipients; n = 1,176) from 2014–2018. Propensity score matching was performed to adjust for baseline differences. Outcomes included 2-year survival from time of treatment or time of listing, freedom from death or delisting for deterioration, and 1-year incidence of major AEs (ie, stroke, renal dysfunction, infection).

**RESULTS:**

After propensity score matching, 2-year survival from treatment was similar for HM3 and HT (88.7% vs 90.2%; HR: 1.18; *P* = 0.53). When comparing outcomes from time of transplant listing vs LVAD implantation, HM3 was associated with higher freedom from death compared with freedom from death or delisting due to deterioration in UNOS (90.1% vs 76.7%; HR: 0.38; *P* < 0.0001). AE analysis showed lower 1-year rates of renal dysfunction requiring dialysis (5.0% vs 10.4%; *P* = 0.016) and fewer infection-related hospitalizations (24.8% vs 34.2%; *P* = 0.012) in HM3 recipients, but a higher incidence of debilitating stroke (3.4% vs 0.3%; *P* = 0.027).

**CONCLUSIONS:**

Contemporary data suggest that durable LVAD therapy may offer survival outcomes comparable to HT in adults <50 years of age. These findings support the consideration of an LVAD-first strategy as a viable initial approach to heart replacement therapy in appropriately selected patients. (Multicenter Study of MagLev Technology in Patients Undergoing Mechanical Circulatory Support Therapy with HeartMate 3 Investigational Device Exemption [MOMENTUM 3 IDE]; NCT02224755) (MOMENTUM 3 Continued Access Protocol [MOMENTUM 3 CAP]; NCT02892955)

## CENTRAL ILLUSTRATION

Outcomes and Adverse Events Following Heart Transplantation and LVAD Implantation in a Cohort of Young Adults

This figure was created with BioRender. HM3 = HeartMate 3; LVAD = left ventricular assist device; MOMENTUM 3 = Multicenter Study of MagLev Technology in Patients Undergoing Mechanical Circulatory Support Therapy with HeartMate 3; UNOS = United Network for Organ Sharing.

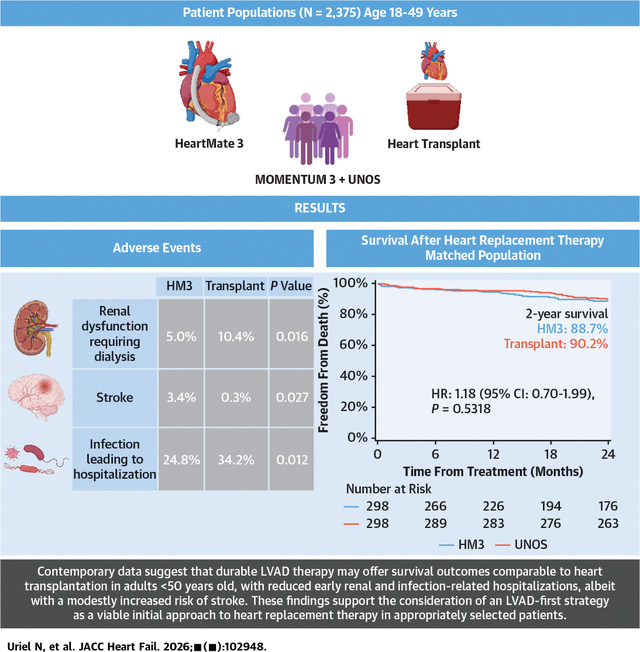

Heart replacement therapy (HRT), encompassing heart transplantation (HT) and durable left ventricular assist devices (LVADs), offers significant improvements in both quality of life and survival for patients with advanced heart failure (HF).^1^ Although HT volumes have increased to more than 4,000 per year in the United States, the growth in individuals with advanced HF has outstripped those gains and has perpetuated the supply-demand mismatch.^2,3^ At the same time, advancements in LVAD technology, particularly with the introduction of the HeartMate 3 (HM3, Abbott), have led to significant improvements in both patient survival and mitigation of adverse events (AEs).^4^ Despite these advancements and the growth in the number of patients with advanced HF, the number of HM3 LVAD implants has not grown proportionately as a result of heart allocation changes enacted in 2018, leading to a marked decrease in their use as a “bridge to transplant” strategy in recent years.^5^

The management of young patients requiring advanced HF therapies presents unique challenges. Given their young age and the finite survival after HT, many of these young patients will require additional HRT during their lives.^6,7^ Options for additional HRT are limited, and the rates of retransplantation remain low at 3%.^8^ Recent reports have noted favorable outcomes for young patients supported by the HM3 LVAD. When focusing on a subgroup of adult patients <50 years of age, patients have demonstrated excellent survival (91.6% at 1 year and 72.6% at 5 years) and a lower incidence of AEs compared with older recipients.^9^

The growing body of evidence regarding the success of the HM3, combined with the complex needs of younger patients who often require multiple interventions, raises a critical question: How can young patients be offered the option of “net prolongation of life?” This requires detailed, informed decision-making with patients as to which HRT (LVAD or HT) would be the best initial therapy. Such discussions need to be comprehensive, including rates of survival and major AEs for both HT and LVAD among a younger age group. To better inform such decision-making, our study compared the outcomes of younger patients (18–49 years of age) with advanced HF who underwent HT compared with those who received the HM3 LVAD.

## METHODS

### STUDY DESIGN.

The MOMENTUM 3 IDE (Multicenter Study of MagLev Technology in Patients Undergoing Mechanical Circulatory Support Therapy With HeartMate 3, Investigational Device Exemption; NCT02224755) trial was a prospective, multicenter, randomized controlled study that demonstrated the HM3 LVAD’s safety and efficacy were superior to the HeartMate II LVAD in patients with advanced HF. The MOMENTUM 3 CAP (Continued Access Protocol; NCT02892955) study was a single-arm study to further assess the HM3 LVAD’s safety and clinical performance. In total, 2,200 patients from 69 centers in the United States received the HM3 LVAD between September 2014 and October 2018 in the MOMENTUM 3 trial, with results previously published.^4,10,11^

The MOMENTUM 3 trial included participants ≥18 years of age, and this analysis focuses on the patients <50 years of age at the time of LVAD implant, alongside the UNOS (United Network for Organ Sharing) registry of adult patients <50 years of age at the time of listing for HT during the same period from 2014–2018. Patients with congenital, infiltrative, or restrictive etiology from each data set were excluded from this analysis. Patients with multiorgan transplantation, retransplantation, or bridged to transplant from LVAD were excluded from the UNOS patient cohort. HM3 patients were analyzed from the time of LVAD implantation, and HT candidates were analyzed separately from 2 distinct time points: from time of transplant or from the time of listing (listed patients may experience adverse outcomes such as death or delisting due to clinical deterioration without receiving a transplant). The HM3 patients were censored after completing 2 years of follow-up evaluation or earlier if they underwent HT, pump explantation, or withdrew from the study. In the primary analysis from time of listing, the primary outcome was freedom from death and delisting due to clinical deterioration. UNOS patients were censored at the time of delisting due to other reasons (eg, being too well). To address potential bias introduced by treating death and delisting due to clinical deterioration as a composite endpoint, a sensitivity analysis was performed in which patients were censored at the time of delisting, regardless of the reason.

Both the MOMENTUM 3 IDE and CAP studies were sponsored by Abbott. The MOMENTUM 3 trial adhered to the principles outlined in the Declaration of Helsinki. All study protocols received approval by each Institutional Review Board, and written informed consent was obtained from each patient or their authorized representative. Studies involving the deidentified UNOS data set have been determined to be exempt from review by the Institutional Review Board of Columbia University Irving Medical Center.

### PROPENSITY SCORE MATCHING.

Baseline characteristics between the HM3 cohort and the UNOS patients at the time of listing and time of transplant differed. Propensity score matching was used to create comparable groups of patients for analysis. Propensity scores were generated using multivariable logistic regression with the HM3 cohort as the treated group. Covariates selected were based on clinical relevance and availability in both the MOMENTUM 3 study and UNOS registry. This included age, mean pulmonary artery (PA) pressure, cardiac output, sex, race, body mass index, estimated glomerular filtration rate, history of diabetes, history of stroke, ischemic or nonischemic etiology, inotrope use (only for analysis start at treatment), and implantable cardioverter-defibrillator. Age and hemodynamic values for the UNOS registry were selected at the time of analysis start, either listing or transplant. No imputations were performed; only patients with complete covariate data were included for propensity score matching. Patients were matched 1:1 using greedy nearest neighbor matching without replacement and a caliper width of 25% of the SD of the logit of the propensity score. Matching was assessed by comparing baseline values using absolute standardized differences.

### ADVERSE EVENTS.

Major AEs were recorded during the MOMENTUM 3 study follow-up period and within 1 year after HT in UNOS. We reported the proportion of patients who experienced major infections leading to hospitalization, renal dysfunction requiring hemodialysis, and debilitating stroke (modified Rankin score >3) within 1 year after HM3 implantation and the proportion of HT recipients in the UNOS registry who experienced hospitalization due to infection, renal dysfunction requiring hemodialysis, and stroke within 1 year after transplant. Major infection in the MOMENTUM 3 trial was defined as a clinical infection accompanied by pain, fever, drainage, and/or leukocytosis that is treated by antimicrobial agents (nonprophylactic) with a positive culture from the infected site unless strong clinical evidence indicates the need for treatment despite negative cultures. Infection in the UNOS registry is defined as hospitalization for infection.

### STATISTICAL ANALYSIS.

Baseline demographic and patient characteristics are presented as mean ± SD for continuous variables and frequency (%) for categorical variables. Covariates were compared before and after propensity score matching using absolute standardized difference (ASD) comparing HM3 to UNOS, with a value <10% considered a good match. Survival analyses were performed using Kaplan-Meier methods and were compared between HM3 and UNOS patients using the log-rank test. HRs for survival analyses were calculated using Cox proportional hazard model. Rate of occurrence of patients experiencing AEs were compared between groups using difference in binomial proportions *z*-test. *P* values are 2-tailed and considered statistically significant at *P* < 0.05. All analyses were performed using SAS 9.4 (SAS Institute).

## RESULTS

### STUDY POPULATION.

**FIGURE 1:**
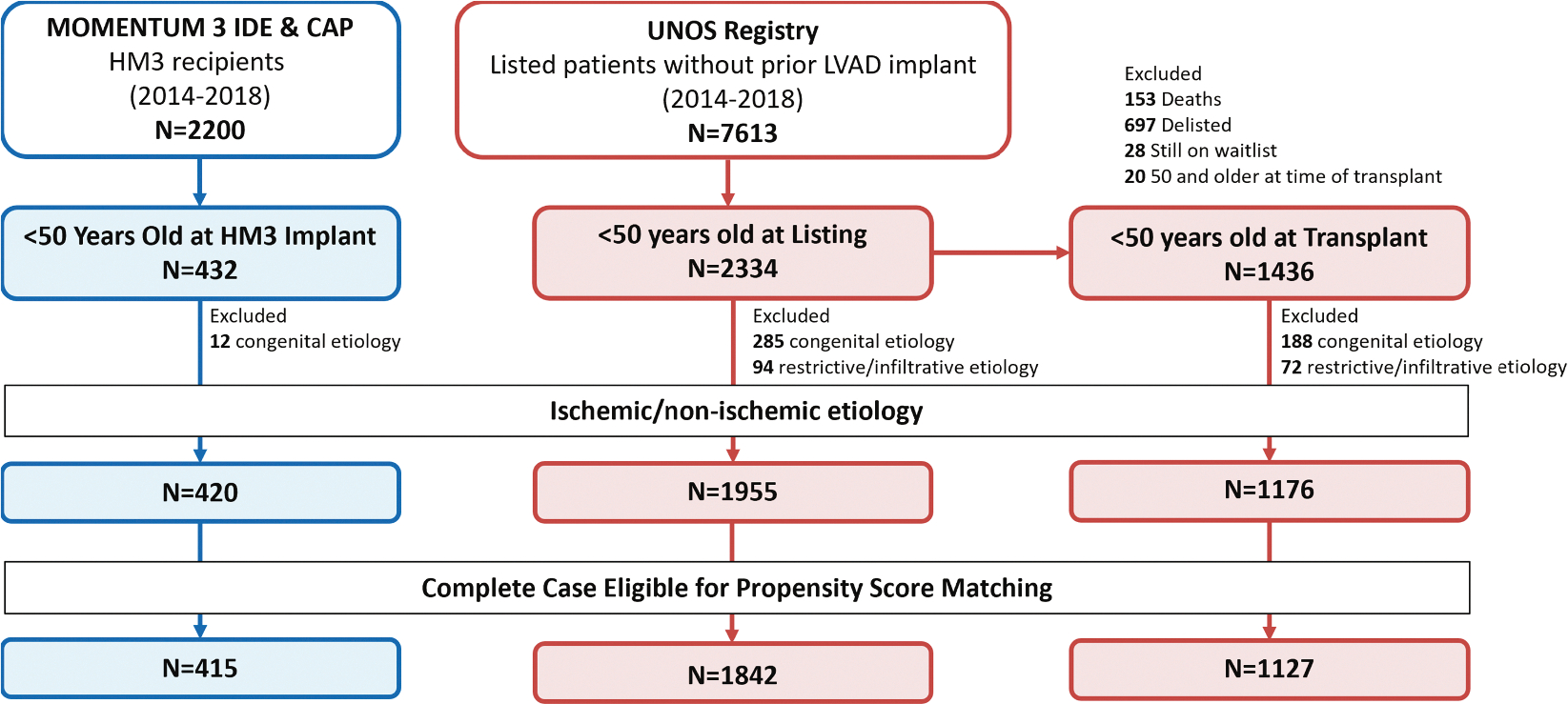
Flow of Patients in the MOMENTUM 3 Trial and UNOS Registry Included in Propensity Score Matching CAP = Continued Access Protocol; HM3 = HeartMate 3; IDE = Investigational Device Exemption; LVAD = left ventricular assist device; MOMENTUM 3 = Multicenter Study of MagLev Technology in Patients Undergoing Mechanical Circulatory Support Therapy with HeartMate 3; UNOS = United Network for Organ Sharing.

A total of 420 adult patients <50 years of age who underwent HM3 LVAD implantation in the MOMENTUM 3 IDE and CAP studies were compared with 1,955 adult patients <50 years of age listed for HT without prior LVAD (and including 1,176 patients who underwent HT before they reached 50 years old) as recorded in the UNOS registry between 2014 and 2018. Figure 1 displays the flow of patients included in this analysis. Patients with congenital, restrictive, or infiltrative cardiomyopathies were excluded from all cohorts. There was minimal data missingness. Approximately 1% of HM3 patients and roughly 5% of both UNOS cohorts were removed from propensity score matching. The baseline characteristics of each complete cases cohort were similar to the overall cohort, indicating no evidence of selection bias (Supplemental Table 1). Two propensity score matching cohorts were developed for comparative analyses: HM3 vs UNOS-listed patients (n = 373 per group) and HM3 vs UNOS HT recipients (n = 298 per group).

The baseline demographic and clinical characteristics before and after propensity matching are summarized in Tables 1 and 2. In the overall cohort, HM3 patients were older (mean age 40.3 vs 38.5 years; ASD: 23.8%), less likely to be female (23.8% vs 37.7%; ASD: 30.6%), and more likely to be Black (44.0% vs 30.0%; ASD: 33%) compared with UNOS-listed patients. Those with HM3 also had a higher prevalence of diabetes (33.1% vs 17.0%; ASD: 37.7%), ischemic etiology (21.2% vs 13.4%; ASD: 20.7%), and higher body mass index (32.6 vs 27.9; ASD: 66.6%) compared with the UNOS-listed patients. HM3 patients also demonstrated higher PA pressures (PA systolic 51.5 vs 42.1 mm Hg; PA diastolic 27.1 vs 21.5 mm Hg; PA mean 36.6 vs 29.5 mm Hg) and a lower estimated glomerular filtration rate (72.1 vs 85.5 mL/min/1.73 m^2^; ASD: 50.8%).

After propensity matching, all major covariates were well balanced between groups with ASD <10%, with the exception of body mass index in the listing cohort comparison (ASD 10.2%) (Table 1). HM3 patients had a higher prevalence of inotrope use (89.0% vs 65.1%, ASD 59.5%) compared with the UNOS patients at the time of transplant (Table 2). Including inotrope use as a covariate, similar balance across all major covariates was achieved in the HM3 vs UNOS transplanted cohorts (Table 2).

### SURVIVAL FROM TIME OF TREATMENT.

**FIGURE 2:**
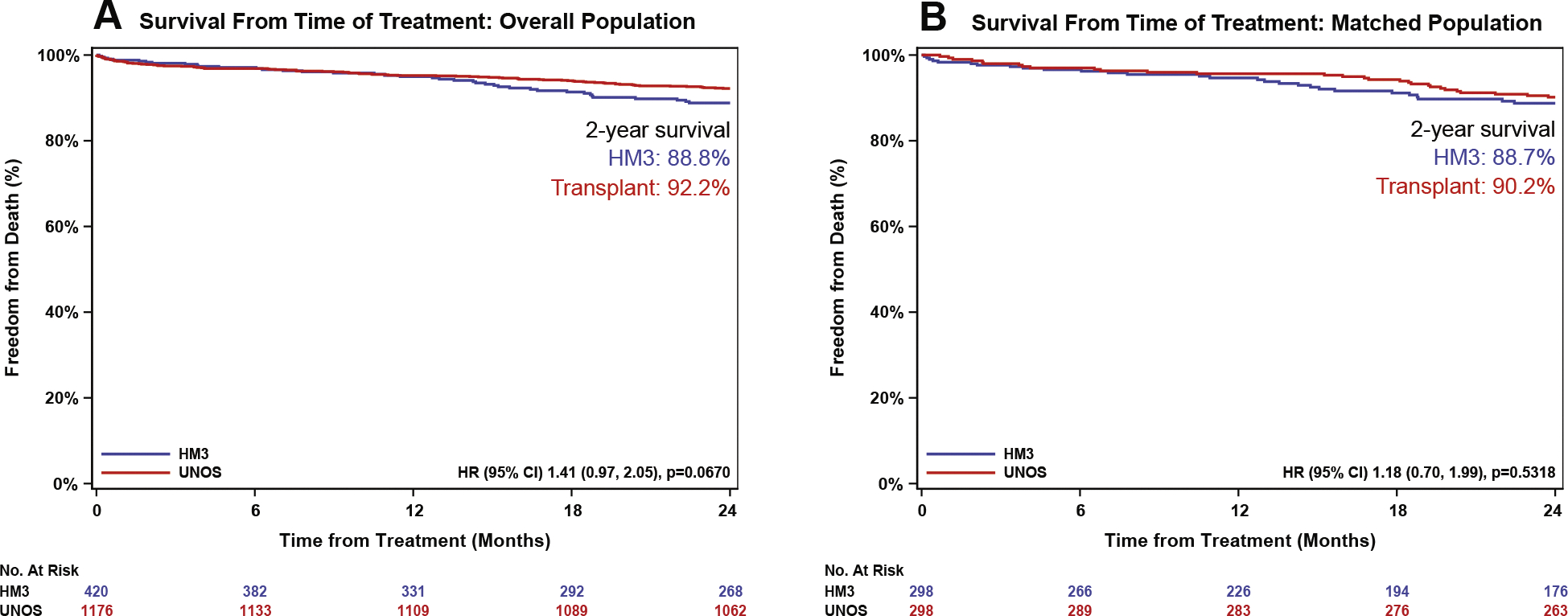
Comparison of 2-Year Survival From the Time of Treatment Between Heart Transplant and HM3 Implantation Overall population (A) and matched populations (B). HRs were calculated using Cox proportional hazards. *P* values were calculated with log-rank test. Abbreviations as in Figure 1.

In the overall cohort analysis of survival from the time of treatment (eg, LVAD implant or HT), the HT recipients had a trend toward higher 2-year survival compared with the HM3 patients. Kaplan-Meier estimated survival at 2 years was 92.2% for HT and 88.8% for HM3 (HR: 1.41 [95% CI: 0.97–2.05]; *P* = 0.067) (Figure 2A). Among 420 HM3 patients, 40 patients (9.5%) died, and 112 patients were censored: 99 (23.6%) were transplanted, 9 (2.1%) were withdrawn or lost to follow-up, and 4 (1.0%) were explanted by 2 years. After propensity matching, survival from time of treatment was nearly identical between the 2 groups. Two-year survival was 88.7% for HM3 recipients and 90.2% for HT recipients (HR: 1.18 [95% CI: 0.70–1.99]; *P* = 0.53) (Figure 2B, Central Illustration).

### SURVIVAL FROM LISTING OR LVAD IMPLANTATION.

**FIGURE 3:**
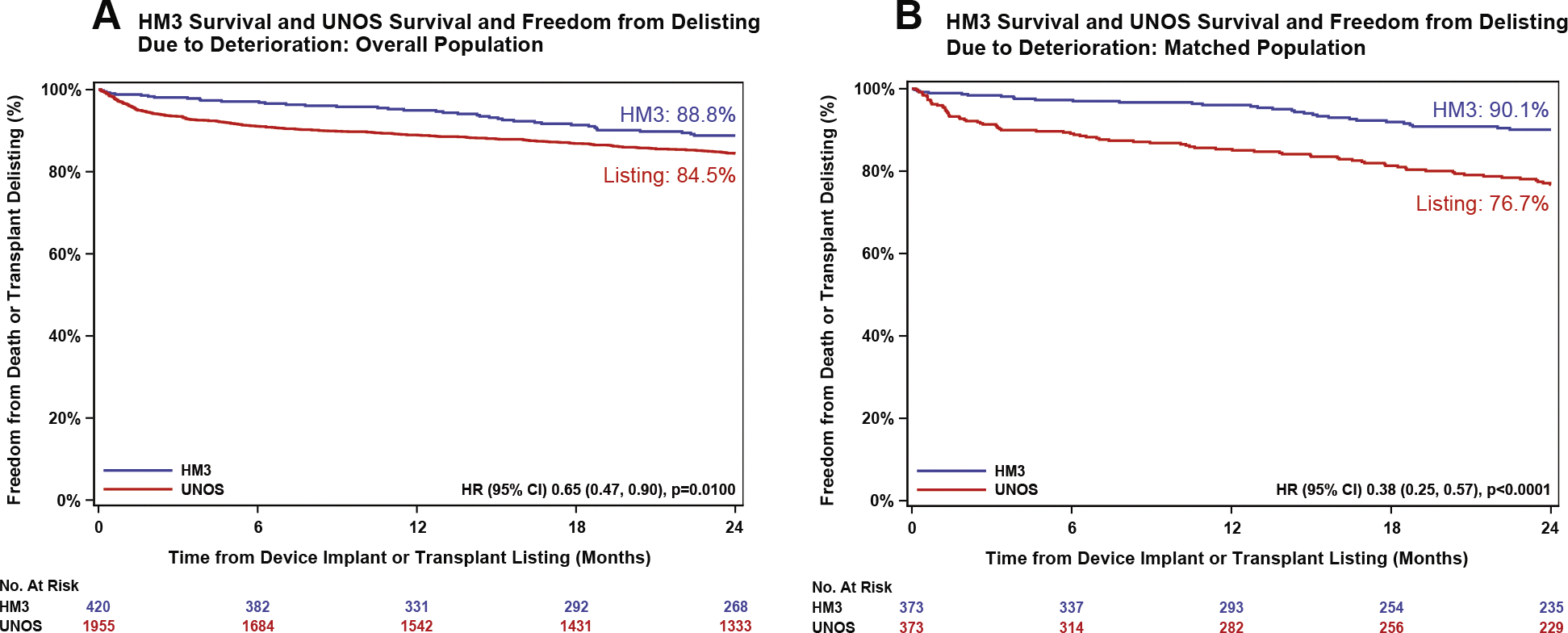
Comparison of Freedom From Death or Delisting Caused by Being Too Sick From UNOS Listing and HM3 Implantation Overall population (A) and matched populations (B). HRs were calculated using Cox proportional hazards. *P* values were calculated with log-rank test. Abbreviations as in Figure 1.

When comparing survival from transplant listing (UNOS) vs LVAD implantation (HM3) in the overall cohort, 2-year freedom from death or delisting due to clinical deterioration was 84.5% for UNOS-listed patients vs 88.8% freedom from death for HM3 patients (HR: 0.65 [95% CI: 0.47–0.90]; *P* = 0.01) (Figure 3A). In the matched cohort, freedom from death or delisting due to clinical deterioration was 76.7% in the UNOS group vs 90.1% freedom from death in the HM3 group (HR: 0.38 [95% CI: 0.25–0.57]; *P* < 0.0001) (Figure 3B).

### SENSITIVITY ANALYSIS.

**FIGURE 4:**
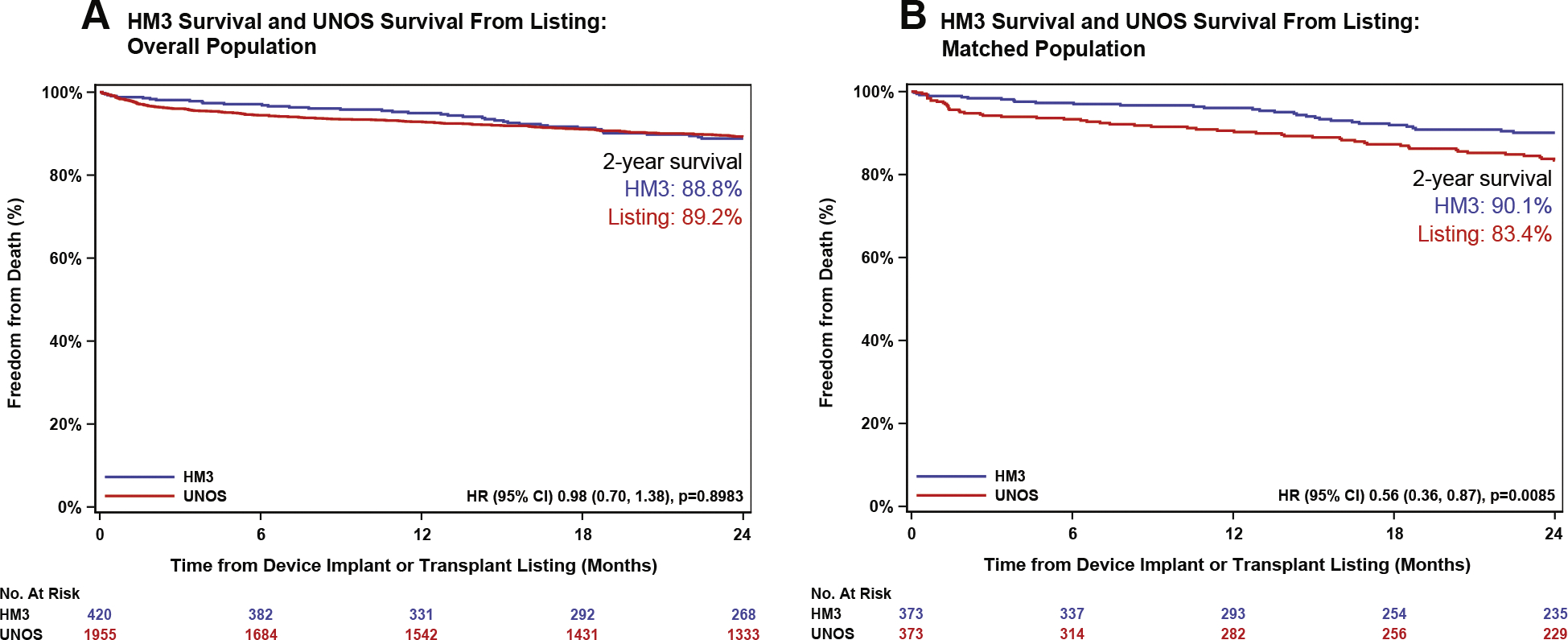
Comparison of Freedom From Death From UNOS Listing and HM3 Implantation Overall population (A) and matched populations (B) while censoring patients who were delisted due to any reasons. HRs were calculated using Cox proportional hazards. *P* values were calculated with log-rank test. Abbreviations as in Figure 1.

To estimate survival after listing for HT, a sensitivity analysis was performed by censoring patients who were delisted for any reason. In the unmatched population, there was no significant difference in 2-year survival between HM3 and UNOS-listed patients (HR: 0.98 [95% CI: 0.70–1.38]; *P* = 0.90) (Figure 4A). In the matched cohort, 2-year survival was significantly higher in HM3 patients compared with patients listed for HT (HR: 0.56 [95% CI: 0.36–0.87]; *P* = 0.009) (Figure 4B).

### POST-TREATMENT ADVERSE EVENTS.

Rates of major AEs occurring within 1 year of treatment are summarized in Table 3. In the unmatched cohort, the incidence of renal dysfunction requiring dialysis was 7.6% in HM3 recipients vs 8.7% in HT recipients (*P* = 0.50). Debilitating stroke occurred in 3.1% of HM3 patients, and stroke occurred in 1.6% of HT patients (*P* = 0.068). Major infections leading to hospitalization occurred in 25.2% of HM3 patients vs 30.5% of HT recipients (*P* = 0.044).

In the propensity-matched population, renal dysfunction requiring dialysis was significantly lower among HM3 patients compared with HT recipients (5.0% vs 10.4%; *P* = 0.016). Conversely, stroke (any stroke [UNOS] vs debilitating stroke [HM3]) occurred more frequently in HM3 patients (3.4%) than HT recipients (0.3%; *P* = 0.027). Rates of major infection requiring hospitalization were lower and statistically significant in HM3 recipients compared with HT recipients (24.8% vs 34.2%; *P* = 0.012).

## DISCUSSION

This study provides a comparative analysis of outcomes in patients <50 years of age who received HM3 LVADs vs those who were listed for or received a HT, using propensity-matched cohorts derived from the MOMENTUM 3 trial and UNOS registry data. Our key findings are: 1) 2-year survival was similar between HM3 recipients and HT recipients; 2) survival and freedom from delisting were significantly lower among patients listed for HT compared with those who received HM3 when analyzed from the time of listing or device implantation; and 3) HM3 patients experienced lower rates of renal dysfunction requiring dialysis and major infections requiring hospitalization within 1 year of treatment but had a higher incidence of stroke. These results suggest that in younger patients eligible for both therapies, an “LVAD-first” strategy may serve as a viable alternative to HT.

HT remains the gold standard for advanced HF, especially in younger patients, with a median survival of 13 years reported in the ISHLT (International Society for Heart and Lung Transplantation) registry.^12^ However, the prevalence of retransplantation remains low, meaning many patients transplanted at a young age may exhaust their HRT options by midlife. By contrast, advances in LVAD technology such as the HM3 magnetically levitated pump have led to improved short- and medium-term survival. Recent data suggest 1- and 5-year survival rates of 85.7% and 59.7%, respectively, with even better outcomes in younger patients (91.6% and 72.6%).^9^ Additionally, the burden of AEs appears more favorable in this subgroup, with 88.8% freedom from stroke and 97% freedom from pump malfunction at 1 year.^9^

Our analysis showed that, in matched cohorts, 2-year survival after HM3 implantation (88.7%) was nearly identical to post-HT survival (90.2%; *P* = 0.53), reinforcing that LVADs can provide comparable outcomes in the short to intermediate term. Moreover, when considering survival from the time of listing (UNOS) vs LVAD implantation, HM3 therapy was associated with significantly lower risk of death compared with risk of death or delisting for deterioration in UNOS (HR: 0.38; *P* < 0.0001). This highlights a major strength of early HM3 use—namely, reducing the risk associated with prolonged waiting periods and the uncertainty of organ availability. Although sensitivity analyses did not show a significant difference in overall mortality when censoring for delisting due to clinical deterioration, the findings reinforce the stability and safety of HM3 support as a bridge to transplant or destination therapy.

Although HT offers meaningful survival, it comes with long-term risks, including graft failure, malignancy, cardiac allograft vasculopathy, and adverse effects of immunosuppression.^13^ Notably, recent registry data show that diabetes mellitus (DM) develops in 21% of patients after transplant, adding to the 22% with preexisting DM. Post-HT DM is strongly associated with worsening renal function and increased mortality (HR: 1.38 for death or retransplantation).^14^ A subset of young transplant recipients also has contraindications for retransplantation due to cumulative comorbidities, further supporting the argument for considering alternatives.

Given the differences in AE reporting in the MOMENTUM 3 trial and UNOS and the lack of well-defined AE definitions in UNOS, we selected comparable events from MOMENTUM 3, including renal dysfunction requiring dialysis, hospitalizations due to major infection, and debilitating stroke, to align as closely as possible with post-transplant AEs such as dialysis, hospitalization due to infection, and stroke reported in UNOS. In our analysis of AEs 1 year after treatment, HM3 recipients experienced significantly fewer cases of renal dysfunction requiring dialysis (5.0% vs 10.4%; *P* = 0.016), possibly reflecting improved renal perfusion and avoidance of calcineurin inhibitors. Conversely, the risk of disabling stroke was significantly higher among HM3 patients (3.4% vs 0.3%; *P* = 0.027), though the absolute event rates were low.

Stroke prevention remains a central challenge in durable LVAD therapy and underscores the importance of careful perioperative screening, meticulous implant technique with stroke mitigation strategies, and robust anticoagulation management. As observed in the recent ARIES-HM3 (Antiplatelet Removal and Hemocompatibility Events With the HeartMate 3 Pump) study, stroke rates after LVAD continue to decline, showing that, with the omission of aspirin, patients experienced 1.9 strokes per 100 patient years of follow-up, with only 0.8 debilitating strokes per 100 patient years.^15^

Rates of major infection requiring hospitalization were lower and statistically significant in the HM3 group (24.8% vs 34.2%; *P* = 0.012). The prevalence of infection in these younger LVAD patients was lower than the overall MOMENTUM 3 cohort,^16^ underscoring the lower rates of AEs in this age group. These event profiles should be thoroughly discussed with younger patients considering HRT because the long-term risks of HT are often underrepresented in counseling.

Importantly, LVAD support combined with guideline-directed medical therapy facilitates myocardial recovery. Specifically, large multicenter studies, including the INTERMACS (Interagency Registry for Mechanically Assisted Circulatory Support) and UNOS, have demonstrated that ~15% of LVAD-supported unselected “all-comers” advanced HF patients improve their left ventricular ejection fraction by an absolute average of 27% (range: 23% to 33%), reaching a left ventricular ejection fraction of >40% with normalization of cardiac dimensions.^17–20^ In selected cohorts (eg, young or middle-aged patients with shorter durations of HF), LVAD-mediated myocardial recovery may be possible in >50%.^21,22^ However, in real-world registry reports, despite these meaningful findings, LVAD weaning for recovery is severely underutilized, taking place extraordinarily less frequently than what would be expected based on the echocardiographic findings reported in these same registry reports.

Noteworthy is a recent multicenter UNOS analysis that demonstrated recovery-based explants were only performed at 38% of transplant programs in the United States whereas durable LVAD weaning for recovery had never occurred in the other 62%.^23^ The investigators concluded that this underutilization is leading to an overall very low incidence at the national level despite a realistic rate being achieved in many other centers for select patients. Importantly, after LVAD weaning, the sustainability of recovery and associated survival is similar to the ISHLT registry post-transplant survival and is accompanied by significant improvements in exercise capacity and quality of life. The generalizability of these findings has been demonstrated through the replication in both single-center and multicenter studies.^18,24–27^

Therefore, the potential decongestion of the transplant wait-list after implementing an LVAD-first strategy has the potential to be: 1) meaningful in absolute numbers; and 2) the duration of this societal benefit will be lasting and not transient. Given that the precious allografts that will be saved will be used by other wait-listed candidates, such an approach can help maximize the societal impact of both HT and LVAD. However, this opportunity may be underutilized in current allocation patterns. A recent study showed significantly reduced rates of recovery compared with the prior allocation system in patients listed for transplant with high urgency (status 1 or 2), possibly due to expedient transplantation, which did not allow sufficient time for potential myocardial recovery.^28^

A key barrier to LVAD-first strategies is the current U.S. allocation system, which prioritizes patients with complications or temporary mechanical support over stable LVAD patients.^29^ It is further compounded by the concern for worse outcomes after HT in those bridged with LVAD compared with those who were on temporary mechanical circulatory support or medical therapy.^30^ As a result, those implanted with LVADs may face prolonged delays in receiving a transplant.^5,31^ Some have proposed modifications to the current system, such as offering upgraded status after predefined time intervals^32^ or applying a “safety net” model similar to that used in dual-organ allocation (eg, kidney after heart, liver, or lung transplant).^33,34^ During the public comment period for the “Escalation of Status for Time on Left Ventricular Assist Device” policy change (active effective June 9, 2025), leaders from HT programs (>60) around the United States submitted a proposal to establish an incentivized bridge to recovery strategy for patients.^35^ The proposed policy would reward those who pursue a durable LVAD with a bridge to recovery strategy (ie, essentially contributing “net positive 1” organ back to the donor pool), providing them with higher wait-list priority if they eventually require HT. This would reward the patients who are contributing to donor organ preservation through LVAD support for those listed on temporary devices, while ensuring them equitable access to HT if needed later.

This analysis adds to the discussion of survival from time of listing, a metric often overlooked. Current listing practices—particularly prolonged use of temporary mechanical circulatory support despite LVAD eligibility—create vulnerabilities in the wait-list population.^36,37^ A recent analysis found that patients with blood type O who were listed with an Impella or intra-aortic balloon pump were at increased risk of post-listing mortality compared with a durable LVAD in the current organ allocation era.^38^ Further, patients may deteriorate while awaiting HT, becoming ineligible for both transplant and LVAD. In our analysis, patients undergoing HM3 implantation had better outcomes than those listed for HT from the time of listing. Stable LVAD patients, supported by current-generation devices, represent a relatively low-risk group who could be safely maintained until suitable donor organs become available, if at all needed.

### STUDY LIMITATIONS.

It is important to note that the selection bias among the HM3 patients due to clinical trial inclusion and exclusion criteria may limit the generalizability of these findings to the broader population.^39^ Additionally, our analysis cohorts only included patients treated between 2014 to 2018, largely prior to the 2018 heart allocation policy change and U.S. Food and Drug Administration approval of the HM3 for long-term support in 2018. As such, the findings may not reflect current practices, given subsequent allocations for HT and advances in LVAD management, and the findings herein should therefore be interpreted within the context of the previous clinical landscape.

Under the new allocation system, although post-transplant survival rates remained stable, overall shorter wait-list times and fewer LVAD patients bridged to transplant highlight the need for future analysis on more contemporary data in this new era. Although the populations were contemporaneous and propensity score matched, residual confounding by unmeasured variables may remain. Differences in data set structures between MOMENTUM 3 (a clinical trial) and UNOS (mandatory self-reporting) may also affect AE reporting; therefore, these results should be interpreted as exploratory rather than as evidence of comparative safety. Additionally, follow-up was limited to 2 years; long-term outcomes including quality of life, device durability, and psychosocial factors warrant further investigation.

Further, outcomes of the HM3 patients who underwent HT were censored at the time of transplant, potentially underestimating the mortality for patients who received a HM3 LVAD before HT but suffered post-transplant mortality. Post-transplant outcomes in LVAD recipients are critical for informing HRT decisions and remain an area for future investigation with extended follow-up periods. Finally, the analysis focused on survival and available AE data, but we were unable to address patient-centered measures of quality of life.

## CONCLUSIONS

This analysis suggests that durable LVAD therapy may offer survival outcomes comparable to HT in adults <50 years of age. These findings support the consideration of an LVAD-first strategy as a viable initial approach to HRT in appropriately selected patients.

## Supplementary Material

MMC1

## Figures and Tables

**TABLE 1:** Baseline Characteristics in the Overall and Matched Populations of HM3 and UNOS Listed Cohort <50 Years of Age

	Overall Population			Matched Population		
		
	HM3 (N = 420)	UNOS Listing (N = 1,955)	ASD,%	HM3 (n = 373)	UNOS Listing (n = 373)	ASD, %

Demographics						
Age, y^a^	40.3 ± 7.2	38.5 ± 8.5	23.8	40.2 ± 7.3	40.6 ± 7.4	4.6
Female	23.8 (100)	37.7 (738)	30.6	24.4 (91)	24.4 (91)	0.0
Race and ethnicity			33.0			4.0
White	45.0 (189)	52.9 (1,035)		46.1 (172)	45.6 (170)	
Black	44.0 (185)	30.0 (586)		42.1 (157)	41.0 (153)	
Asian	0.0 (0)	0.0 (0)		0.0 (0)	0.0 (0)	
Hispanic	6.4 (27)	11.7 (228)		6.7 (25)	7.8 (29)	
Other	4.5 (19)	5.4 (106)		5.1 (19)	5.6 (21)	
Medical history and previous treatment						
Diabetes	33.1 (139)	17.0 (333)	37.7	30.3 (113)	30.6 (114)	0.6
Ischemic etiology	21.2 (89)	13.4 (262)	20.7	20.4 (76)	20.9 (78)	1.3
Previous stroke	7.6 (32)	6.2 (121)	5.6	7.2 (27)	6.7 (25)	2.1
Previous implantation of defibrillator	66.9 (281)	73.6 (1,439)	14.7	68.1 (254)	67.3 (251)	1.7
Previous inotrope use^b^	89.0 (374)	64.5 (769/1,192)	60.7	89.5 (334)	71.5 (143/200)	46.8
Baseline characteristics						
BMI, kg/m^2^	32.6 ± 8.2	27.9 ± 5.6 (1,952)	66.6	31.3 ± 7.3	30.6 ± 5.6	10.2
eGFR, mL/min/1.73 m^2^	72.1 ± 26.0	85.5 ± 26.8	50.8	74.0 ± 26.2	72.8 ± 25.4	4.6
PA systolic pressure, mm Hg	51.5 ± 14.8 (417)	42.1 ± 13.6 (1,912)	66.5	50.0 ± 14.3	49.8 ± 14.8	1.1
PA diastolic pressure, mm Hg	27.1 ± 9.3 (417)	21.5 ± 8.7 (1,911)	62.2	26.0 ± 8.8	26.8 ± 9.3	7.9
PA mean pressure, mm Hg	36.6 ± 10.9 (416)	29.5 ± 10.2 (1,891)	67.5	35.4 ± 10.5	35.9 ± 10.7	4.2
Cardiac output, L/min	4.10 ± 1.38 (416)	4.10 ± 1.28 (1,866)	0.4	4.06 ± 1.40	3.99 ± 1.18	5.1

Values are mean ± SD, % (n), or mean ± SD (n), unless otherwise indicated.

a Age at the time of listing for UNOS listing cohort.

b Previous inotrope use at the time of transplant for UNOS listed patients who received a heart transplant.

ASD = absolute standardized difference; BMI = body mass index; eGFR = estimated glomerular filtration rate; HM3 = HeartMate 3; PA = pulmonary artery; UNOS = United Network of Organ Sharing.

**TABLE 2:** Baseline Characteristics in the Overall and Matched Populations of HM3 and UNOS Cohort Who Received a Heart Transplant <50 Years of Age

	Overall Population			Matched Population		
		
	HM3 (N = 420)	UNOS Transplant (N = 1,176)	ASD, %	HM3 (n = 298)	UNOS Transplant (n = 298)	ASD, %

Demographics						
Age, y^a^	40.3 ± 7.2	38.4 ± 8.5	24.6	40.2 ± 7.4	39.7 ± 7.8	6.8
Female	23.8 (100)	40.2 (473)	35.7	25.5 (76)	25.5 (76)	0.0
Race			36.6			9.1
White	45.0 (189)	54.1 (636)		49.7 (148)	50.7 (151)	
Black	44.0 (185)	28.2 (332)		38.6 (115)	35.9 (107)	
Asian	0.0 (0)	0.0 (0)		0.0 (0)	0.0 (0)	
Hispanic	6.4 (27)	12.2 (143)		6.4 (19)	7.7 (23)	
Other	4.5 (19)	5.5 (65)		5.4 (16)	5.7 (17)	
Medical history and previous treatment						
Diabetes	33.1 (139)	14.5 (171)	44.6	25.5 (76)	24.2 (72)	3.1
Ischemic etiology	21.2 (89)	11.6 (137)	26.0	18.8 (56)	19.1 (57)	0.9
Previous stroke	7.6 (32)	5.8 (68)	7.4	7.4 (22)	6.4 (19)	6.2
Previous implantation of defibrillator	66.9 (281)	75.3 (886)	18.7	66.8 (199)	70.8 (211)	8.7
Previous inotrope use	89.0 (374)	65.1 (765)	59.5	86.6 (258)	85.6 (255)	2.9
Baseline characteristics						
BMI, kg/m^2^	32.6 ± 8.2	26.9 ± 5.2 (1,174)	83.4	30.1 ± 7.2	29.8 ± 5.0	4.6
eGFR, mL/min/1.73 m^2^	72.1 ± 26.0	86.5 ± 26.0	55.5	76.3 ± 26.7	78.0 ± 24.8	6.8
PA systolic pressure, mm Hg	51.5 ± 14.8 (417)	40.8 ± 13.8 (1,162)	75.1	48.1 ± 13.9	47.8 ± 14.2	2.1
PA diastolic pressure, mm Hg	27.1 ± 9.3 (417)	20.8 ± 8.7 (1,161)	70.3	24.8 ± 8.5	25.2 ± 8.7	5.3
PA mean pressure, mm Hg	36.6 ± 10.9 (416)	28.6 ± 10.5 (1,149)	74.9	33.9 ± 10.1	34.2 ± 11.2	2.5
Cardiac output, L/min	4.10 ± 1.38 (416)	4.30 ± 1.47 (1,142)	13.8	4.11 ± 1.46	4.00 ± 1.25	8.4

Values are mean ± SD, % (n), or mean ± SD (n), unless otherwise indicated.

a Age at the time of transplant for UNOS transplant cohort.

Abbreviations as in Table 1.

**TABLE 3:** Comparison of Adverse Events Between HM3 and Heart Transplant Cohorts Within 1 Year After Treatment

	HM3		UNOS Transplant		
			
	n/N	Proportion (95% CI)	n/N	Proportion (95% CI)	*P* Value

Renal dysfunction requiring dialysis					
Overall population	32/420	7.6 (5.4–10.6)	102/1,176	8.7 (7.2–10.4)	0.50
Matched population	15/298	5.0 (3.1–8.2)	31/298	10.4 (7.4–14.4)	0.016
Stroke^a^					
Overall population	13/420	3.1 (1.8–5.3)	19/1,176	1.6 (1.0–2.5)	0.068
Matched population	10/298	3.4 (1.8–6.1)	1/298	0.3 (0.0–2.3)	0.027
Major infection leading to hospitalization^b^					
Overall population	106/420	25.2 (21.3–29.6)	349/1,176	30.5 (27.9–33.2)	0.044
Matched population	74/298	24.8 (20.3–30.1)	101/298	34.2 (29.0–39.8)	0.012

a Stroke for HM3 was defined as debilitating stroke (modified Rankin score >3). Stroke for UNOS was any stroke.

b Infections for HM3 were defined as major infections requiring hospitalization. Infections for UNOS were defined as hospitalizations due to infection.

Abbreviations as in Table 1.

## PERSPECTIVES

### COMPETENCY IN MEDICAL KNOWLEDGE:

Survival for younger adults (18–49 years of age) with advanced heart failure is similar after heart transplant or left ventricular assist device placement.

### TRANSLATIONAL OUTLOOK:

Individualized shared decision-making is necessary to determine the best approach for each patient, weighing the strengths and limitations of each advanced heart failure therapy.

## ABBREVIATIONS AND ACRONYMS

AE: adverse event
ASD: absolute standardized difference
DM: diabetes mellitus
HF: heart failure
HRT: heart replacement therapy
HT: heart transplantation
LVAD: left ventricular assist device
PA: pulmonary artery
