# Supplementary material for: Heart Replacement Therapy in Young Patients: A Comparative Analysis of HeartMate 3 LVAD and Heart Transplant Using MOMENTUM 3 and UNOS Registry
Source: JACC Heart Fail. Author manuscript; Available in PMC 2026 Mar 14. (PMC12987674; doi:10.1016/j.jchf.2026.102948)
Supplement: MMC1 [file NIHMS2145052-supplement-MMC1.docx]

**SUPPLEMENTAL TABLE 1** Baseline Characteristics in the HM3, UNOS Transplant and Listing Cohorts Under 50 Years of Age With Complete Case

|  | **Complete Case** | | |
| --- | --- | --- | --- |
|  | **HM3 (N = 415)** | **UNOS Listing**  **(N = 1,842)** | **UNOS Transplant (N = 1,127)** |
| **Demographics** |  |  |  |
| Age, y^a^ | 40.3 ± 7.2 | 38.7 ± 8.4 | 38.4 ± 8.5 |
| Female sex | 24.1% (100) | 37.8% (696) | 39.8% (449) |
| Race and ethnicity |  |  |  |
| White | 44.6% (185) | 53.2% (980) | 54.0% (609) |
| Black | 44.3% (184) | 29.9% (551) | 28.2% (318) |
| Asian | 0.0% (0) | 0.0% (0) | 0.0% (0) |
| Hispanic | 6.5% (27) | 11.6% (214) | 12.2% (138) |
| Other | 4.6% (19) | 5.3% (97) | 5.5% (62) |
| **Medical History and Previous Treatment** |  |  |  |
| Diabetes | 33.3% (138) | 17.6% (324) | 14.6% (165) |
| Ischemic etiology | 21.2% (88) | 13.0% (239) | 11.5% (130) |
| Previous stroke | 7.5% (31) | 6.2% (115) | 5.9% (66) |
| Previous implantation of defibrillator | 67.2% (279) | 74.9% (1,379) | 75.1% (846) |
| Previous inotrope use^b^ | 88.9% (369) | 65.2% (740/1,135) | 65.1% (734) |
| **Baseline Characteristics** |  |  |  |
| Body mass index, kg/m^2^ | 32.5 ± 8.1 | 28.0 ± 5.5 | 26.9 ± 5.2 |
| eGFR, mL/min/1.73 m^2^ | 72.3 ± 25.9 | 85.1 ± 26.6 | 86.5 ± 26.1 |
| PA systolic pressure, mm Hg | 51.5 ± 14.8 | 42.1 ± 13.6 (1,840) | 40.8 ± 13.8 (1,126) |
| PA diastolic pressure, mm Hg | 27.1 ± 9.3 | 21.5 ± 8.6 (1,840) | 20.8 ± 8.7 (1,126) |
| PA mean pressure, mm Hg | 36.6 ± 10.9 | 29.5 ± 10.1 | 28.6 ± 10.5 |
| Cardiac output, L/min | 4.09 ± 1.38 | 4.10 ± 1.28 | 4.29 ± 1.47 |
| Categorical variables are listed as percentage and frequency, and continuous variables as mean ± SD (n).  ^a^Age at the time of transplant for UNOS transplant cohort or time of listing for UNOS listing cohort.  ^b^Previous inotrope use at the time of transplant for UNOS patients who received a heart transplant.  ASD = absolute standardized difference; eGFR = estimated glomerular filtration rate; HM3 = HeartMate 3; PA = pulmonary artery; UNOS = United Network of Organ Sharing. | | | |
